# Molecular features of influenza A (H1N1)pdm09 prevalent in Mexico during winter seasons 2012-2014

**DOI:** 10.1371/journal.pone.0180419

**Published:** 2017-07-10

**Authors:** Rocío Arellano-Llamas, Luis Alfaro-Ruiz, Cristian Arriaga Canon, Ivan Imaz Rosshandler, Alfredo Cruz-Lagunas, Joaquín Zúñiga, Rosa Rebollar Vega, Christopher W. Wong, Sebastian Maurer-Stroh, Sandra Romero Córdoba, Edison T. Liu, Alfredo Hidalgo-Miranda, Joel A. Vázquez-Pérez

**Affiliations:** 1 Instituto Nacional de Medicina Genómica, Mexico City, Mexico; 2 CONACYT- Instituto Nacional de Cancerología, Mexico City, Mexico; 3 Instituto Nacional de Enfermedades Respiratorias Ismael Cosío Villegas, Mexico City, Mexico; 4 Genome Insitute of Singapore, Singapore, Singapore; 5 Bioinformatics Institute of Singapore, Singapore, Singapore; 6 The Jackson Laboratory, Bar Harbor, Maine, United States of America; The Scripps Research Institute, UNITED STATES

## Abstract

Since the emergence of the pandemic H1N1pdm09 virus in Mexico and California, biannual increases in the number of cases have been detected in Mexico. As observed in previous seasons, pandemic A/H1N1 09 virus was detected in severe cases during the 2011–2012 winter season and finally, during the 2013–2014 winter season it became the most prevalent influenza virus. Molecular and phylogenetic analyses of the whole viral genome are necessary to determine the antigenic and pathogenic characteristics of influenza viruses that cause severe outcomes of the disease. In this paper, we analyzed the evolution, antigenic and genetic drift of Mexican isolates from 2009, at the beginning of the pandemic, to 2014. We found a clear variation of the virus in Mexico from the 2011–2014 season due to different markers and in accordance with previous reports. In this study, we identified 13 novel substitutions with important biological effects, including virulence, T cell epitope presented by MHC and host specificity shift and some others substitutions might have more than one biological function. The systematic monitoring of mutations on whole genome of influenza A pH1N1 (2009) virus circulating at INER in Mexico City might provide valuable information to predict the emergence of new pathogenic influenza virus

## Introduction

Since the emergence of the pandemic H1N1pdm09 virus in Mexico and California, biannual increases in the number of cases have been detected in Mexico [1, 2]

The first outbreak began from late March to July 2009, followed by a second wave from late August 2009 to March 2010 [1]. The 2010–2011 winter season was characterized by low cases of pandemic virus and a predominance of Influenza A H3N2 and Influenza B [3]. As observed in previous seasons, pandemic A/H1N1 09 virus was detected in severe cases during the 2011–2012 winter season and finally during the 2013–2014 winter season it became the most prevalent influenza virus [3]. Increased cases of pneumonia and hospitalizations were observed at the National Institute for Respiratory Diseases in Mexico City in these seasons and these cases were related to the presence of the pandemic A/H1N1 virus [4].

Molecular and phylogenetic analyses of the viral genome are necessary to determine the antigenic and pathogenic characteristics of influenza viruses that cause severe outcomes of the disease [5]. In this context, one of the first studies in Mexico that analyzed sequences of HA and NA from pandemic A/H1N1 virus was done in early days of the pandemic outbreak in 2009 to detect and monitor pathogenicity and resistance substitutions affecting the outcome or severity of the respiratory diseases [6]. Later, oseltamivir-resistant virus was observed, showing low rates of the H275Y substitution [7]. With regards to pathogenicity, only the D222G/N/S substitutions were associated with severity and mortality in patients from Mexico City. Other mutations associated with adaptation or increased pathogenicity in human or avian influenza virus such as E627K in PB2 or T123V in NS1 were not detected [8]. The introduction of different substitutions in other segments, particularly the HA gene in segment 4, might affect the immunogenicity of the pandemic virus. Importantly, the strain recommended by the World Health Organization (WHO) for vaccine composition remained the A/California/7/2009 until the 2016–2017 influenza season (northern hemisphere winter), suggesting that the pandemic virus has not undergone antigenic changes during several years of circulation. Antigenic drift is caused by mutations in the antigenic sites adjacent to the receptor binding site of HA. This is why monitoring the molecular and functional properties of HA becomes essential for effective vaccine development. Our previous data showed that substitutions near the immunogenic domain in HA appeared since 2012, probably compromising the effectiveness of vaccination [8]. Few reports of HA pandemic virus characterization during the 2014 season have been reported and have also identified substitutions that might affect a region of HA targeted by antibodies in middle-aged adults. Recently, an extensive study about the global evolution of pandemic influenza was reported, however only few whole HA sequences from Mexico were included [9]. Furthermore, during the 2015–2016 influenza season, a delay in the wave of pandemic A/H1N1 confirmed cases was observed and until now in March 2016, a significant increase in the severity of cases in Mexico City hospitals has been observed.

In this paper, we analyzed the evolution, antigenic and genetic drift of Mexican isolates from 2009, at the beginning of the pandemic to 2014.

## Materials and methods

### Viral samples isolation from hospitalized patients

Clinical samples from patients with influenza-like illness were collected, at the INER as part of routine care and diagnosis. Samples were collected through nasopharynx swabs or bronchial aspiration. These clinical samples were typed using specific primers for influenza H3N2, B and A/H1N1pdm09. Samples positive for the influenza AH1N1pdm09 virus were used for RNA extraction, from 200 ul of the sample. The Invisorb extraction columns were used for samples obtained through nasal swabs, (Invisorb spin virus mini kit, Cat No.1040300200). Viral typing was performed by rtPCR and according to the Center of Disease Control (CDC) guidelines.

Approval for this study was obtained from the Ethics and Research Institutional Boards of the National Institute of Genomic Medicine and the Ethics and Research Boards of the National Institute of Respiratory Diseases. Informed consent was provided according to the Declaration of Helsinki. Written informed consent was obtained from all studied patients and/or from their relatives or authorized legal representative.

### Viral genome sequencing

50 clinical samples were positive for AH1N1pdm09 and we obtained the whole viral genome sequence in 23 of these samples. 13 samples were sequenced using high-density oligonuclotides microarrays and 10 samples were sequenced using massive parallel sequencing with the Illumina platform. 4 samples were sequenced using both technologies.

Additionally, there were 4 samples where we could not achieve whole genome sequences and we only obtained sequences from specific segments (INMEGEN-INER 1, NP, M, NS; INMEGEN-INER 7, PB2, NP, M, NS; INMEGEN-INER 16 and 17, all segments except HA)

### Re-sequencing array

The whole genome sequences from clinical samples were obtained using high-density oligonuclotides microarrays for re-sequencing, (GIS H1N1 Flu BioSurveillance Array Nimblechip 132k) as previously described [10]

Retro transcription (RT) of the influenza AH1N1pdm09 viral genome was carried out from RNA extracted from nasal swabs of clinical samples positive to this viral type. We used the Invitrogen Super Script III high fidelity enzymes kit (SuperScript III First strand synthesis system for RT-PCR, Cat No. 18080–051) and primers from the Biosurveillance Resequencing Oligo Kit (AITbiotech, AITBH1N1M-50), for RT-PCR. Later we used 2 ul of cDNA from the RT for the 8 segments amplification by PCR with Platinum Taq DNA polymerase (Platinum Taq DNA polymerase, Thermo Scientific, Foster City, CA, USA) and cocktail of primers included in the resequencing oligo kit. PCR conditions were 94°C, 2min; 40 cycles (94°C, 30 s; 66°C, 3min); 72°C, 5min.

PCR fragments from each sample were pooled and labeled with CY3 (Nimbelgen One-color DNA Labeling Kit, REF 05 223 555 001) independently and 800 ng of the labeled reaction were used for array hybridization, following the manufacturer´s instructions (NimbleGen Hybridization Kit, REF 05 583 683 001).

Microarrays were scanned using a GenePix 4000B scanner and processed with the NimbleScan and EvolStar softwares to translate the fluorescence intensity into nucleotides and obtain a sequence FASTA file.

In total, the array contains 8,236 control probes and 121,928 H1N1(2009) probes, which provides 2X coverage of the entire H1N1(2009) genome, and up to 8X coverage of the regions comprising the 36 mutation hotspots and 10 drug-binding sites [11].

Another set of 10 clinical samples were sequenced using massive paralleled sequencing (MPS) on an Illumina MiSeq (Illumina, CA, USA), as described below. Four samples were sequenced in parallel by microarrays and (MPS).

#### Massive parallel sequencing

The 8 viral segments were amplified simultaneously and directly from clinical samples, using MBTuni12 and MBTuni13 primers, as described elsewhere [12]. Amplification products for 10 samples were gel-purified (QIAquick Gel Extraction Kit; QIAGEN, Valencia, CA). Barcoded libraries for NGS were produced using Nextera XT DNA Library Preparation kit and paired-end sequencing (2x250 cycles) was performed using the MiSeq platform (Illumina). The reads were mapped to A/California/07/2009(H1N1)/2009(H1N1), data was obtained from the NIAID Influenza Research Database (IRD) [13] (access number of this sequence is presented in S1 Table) using SMALT V.0.7.6. Assembly was performed using Velvet package (Velvet V1.2.10) and visualized with TABLET (V 1.15.09.01). A 100% coverage was achieved for each virus, with at least 90 depth for HA, NS, NA, M and NP segments (mean coverage: 10). The PA, PB1 and PB2 segments had 80% coverage at >10x. The sequences from this study were deposited at the NIAID Influenza Research Database (IRD, http://www.fludb.org) [13] and are available in GenBank (Accesion numbers can be found in S1 Table)

### Mutation analysis

The sequences were analyzed using A/California/07/2009(H1N1)/2009 amino acid sequence as reference and compared to global sequences at FluSurver [14] from 2009 to 2016, this for each of the eight viral segments (Access number in S1 Table), in order to identify novel substitutions and determine how many times reported substitutions have been previously reported. Sequences were aligned using the CLUSTAL W method in Molecular Evolutionary Genetics Analysis (MEGA 6.0) software [15].

### Analysis of HA antigenic sites

The analysis of substitutions at HA was carried out using A/California/07/2009(H1N1)/2009 as the reference sequence, to establish changes at the antigenic sites and in neighboring positions for the clinical samples sequenced from years 2012 to 2014. Substitutions were obtained by manual search at aligned sequences in MEGA 6.0.

### Phylogeny

Full-length nucleotide sequences were selected for all segments of human influenza H1N1 viruses reported between 2011 and December 31, 2014 in IRD, including genomes that had all corresponding segments. We included the ten whole genome sequences (2013–2014) from this study for the analyses of all 8 viral segments and 20 sequences of PB2, 18 of PB1, 17 of PA, 20 of HA, 21 of NP, 18 of NA, 27 of M and 23 of NS from clinical samples collected during 2011 and 2012; 12 of these samples had whole genome sequence data. Sequence alignments were created by Multiple Sequence Comparison by Log-Expectation (MUSCLE) [16] and edited by MEGA 6.0. A maximum likelihood tree was constructed for each influenza segment using MEGA 6.0. The Tajima-Nei model was selected with 5-parameter gamma distributed rates and 1,000 bootstrap replicates. Editing of trees was made using Tree (http://tree.bio.ed.ac.uk/software/figtree/).

## Results

Clinical and demographic characteristics of the studied individuals are summarized in Table 1. The mean age of the patients was 45.4 ± 20.8 years and 76.4% of these individuals were male. The mean BMI (kg/m^2^) was 29.2, and 23.5% required mechanical ventilation for 15.2 (mean) days. Patients had 22 days of hospitalization stay, 17.6% of them had co-morbidities such as asthma and chronic obstructive pulmonary disease (COPD), and 5.8% of these patients died. Patients who required mechanical ventilation displayed a Kirby index (PaO_2_/FiO_2_) (mean: 104.7). The main signs and symptoms at the start of the illness included fever, myalgia, cough, and headaches, while chest pain, dyspnea, and cyanosis occurred after the third day. Critically ill patients received oseltamivir (150 mg/day) during the period while they were under mechanical ventilation while non-critically ill patients received 150mg/day of oseltamivir for 5 days.

**Table 1:** Clinical and demographic characteristics of the studied patients.

Variable	A/H1N1 Patients
Age mean (±SD)	45.46 ± 20.8
Gender Male (%)	76.4
BMI mean (kg/m^2^)	29.2
Mechanical Ventilation (%)	23.5
Mechanical ventilation mean (days)	15.2
Hospitalization (days)	22 ± 8
Co-morbidities* (%)	17.6
Fatal outcome (%)	5.8

Data are means ± standard deviation (SD), or number and percentage.

*2 patients had asthma and 1 COPD.

### Substitutions detected by whole genome sequencing of the AH1N1 09pdm influenza virus

Substitutions observed in the whole genome of the pandemic A/H1N1 virus were classified by viral gene segment, year of isolates and description of the mutation. The analysis of the sequences for each of the eight viral segments was performed using the A/California/07/2009(H1N1)/2009 strain amino acid sequence as reference and comparing global sequences at FluSurver from 2009 to 2016. Table 2 shows the substitutions detected for each of the 8 viral segments corresponding to the viral genes (HA, NP, NA, M1/M2, NS1/NS2, PB2, PB1, and PA), the corresponding year of detection (*italics* for 2011–2012 and **bold** for 2013–2014), and its potential biological effect.

**Table 2:** Substitutions observed in pandemic A/H1N1 virus strains classified by gene segment, year of circulation and description.

Gen	Substitution	Biological Effect	Times reported	Reference**
**PB2**	*T76I*	Uncharacterized	14	
	*G128C*	Uncharacterized	2 (Reported once before)	
	*V227A*	Uncharacterized	3 (First detected in mexico)	
	*R591P*	Virulence, effective replication	First time reported	[28]
	**V613A**	Host specificity shift	3	[28], [37]
	**V640M**	BSL*	First time reported	[34]
	**G644R**^a^	**BSL/VOI**	4	[28], [46]
	**T676I**^a^	Adaptation	12	[17]
**PB1**	*Q569K*	Uncharacterized	First time reported	
	*S704T*	T cell epitope presented by MHC	First time reported	[29], [30]
	**G154A**	Uncharacterized	First time reported	
	**E177Q**	Uncharacterized	First time reported	
	**E178K**	Uncharacterized	4	
	**M339T**	Uncharacterized	2 (Reported once before)	
**PA**	*L109F*	**BSL**	First time reported	[32], [33] Near to an importance región
	*T151N*	**BSL/VOI**	5	
	*T162P*	VOI*	First time reported	
	*E623D*	**BSL/VOI**	3	[47]
	**I13V**^a^	**VOI**	16	[38]
	**F35L**	**BSL**	12	[41]
	**E298K**	Uncharacterized	9	
**HA**	*H25R*	**BSL/VOI**/BHP/Ab’s* Recognition	2 (Reported once before)	[43] Near to an importance region
	*E373G*	**BSL/VOI**/Ab’s Recognition	19	[42], [43]
	**V7I**^a^	Uncharacterized	22	
**NP**	*E11G*	Uncharacterized	First time reported	
	*A27V*	Uncharacterized	2 (Reported once before)	
	*Y281C*	Uncharacterized	First time reported	
	**G356R**	**VOI**/BNA*	First time reported	[35], Importance region
	**S482N**	**VOI**	10	
**NA**	*I122V*	Uncharacterized	11	
	*V177I*	**BSL/VOI**	4	[45] Near to an importance region
	**D199N**	Antigenic drift/Ab’s Recognition	30	[44], [45]
	**R220G**	**VOI**/Ab’s Recognition	13	[45] Near to an importance region
	**Q308L**^a^	Uncharacterized	6	
	**T452I**^a^	**BSL**/BHP*	18	[49]
**M1**	*G18S*	**VOI**	2	[31]
	*N207S*	Uncharacterized	11	
	**A33T**	**VOI**	6	[31]
	**M165L**	Uncharacterized	2 (Reported once before)	[31]
**M2**	**E16A**	Host Specificity shift	First time reported	
**NS1**	*G45R*	**BNA**	10	[50]
	*A122T*	**VOI**	18	[39], [40] Near to an importance región
	**L36I**^a^	**BSL/VOI**	6	[48]
	**Q63K**	**BSL/VOI**	First time reported	[48], [36]
	**S74N**	**BSL/VOI**/BHP	11	[48], [40]
	**N127S**^a^	**BSL/VOI**	5	[48]
	**L185F**	Uncharacterized	9	

This data were obtained submitting FASTA sequences to FluSurver

*Abreviations: **BSL**, binding small ligands; **VOI**, viral oligomerization interfaces; BHP, binding host proteins; **BNA**, binding nucleic acids; Ab’s, antibodies.

^a^ Two or more sequences

** The functional annotations of sites and mutations in the list here are not purely predicted computationally but are in fact strictly based on experiments

13 of the substitutions we identified were novel, and their potential biological role has not been defined. Substitutions that have been previously reported are involved in host specificity shift, viral oligomerization interfaces (**VOI**), binding small ligands (**BSL**), Ab’s recognition, binding host proteins (**BHP**), binding nucleic acids (**BNA**) and antigenic drift. **VOI** is calculated automatically from known structures and captures sites both in viral oligomerization sites as well as crystal contacts. BHP includes mostly interaction with human immune response proteins. BSL is derived from small ligands seen in known crystal structures and, in case of influenza surface proteins, often signifies interaction with small glycans which can be in or near a glycosylation site.

### Phylogenetic analysis

**Fig 1:**
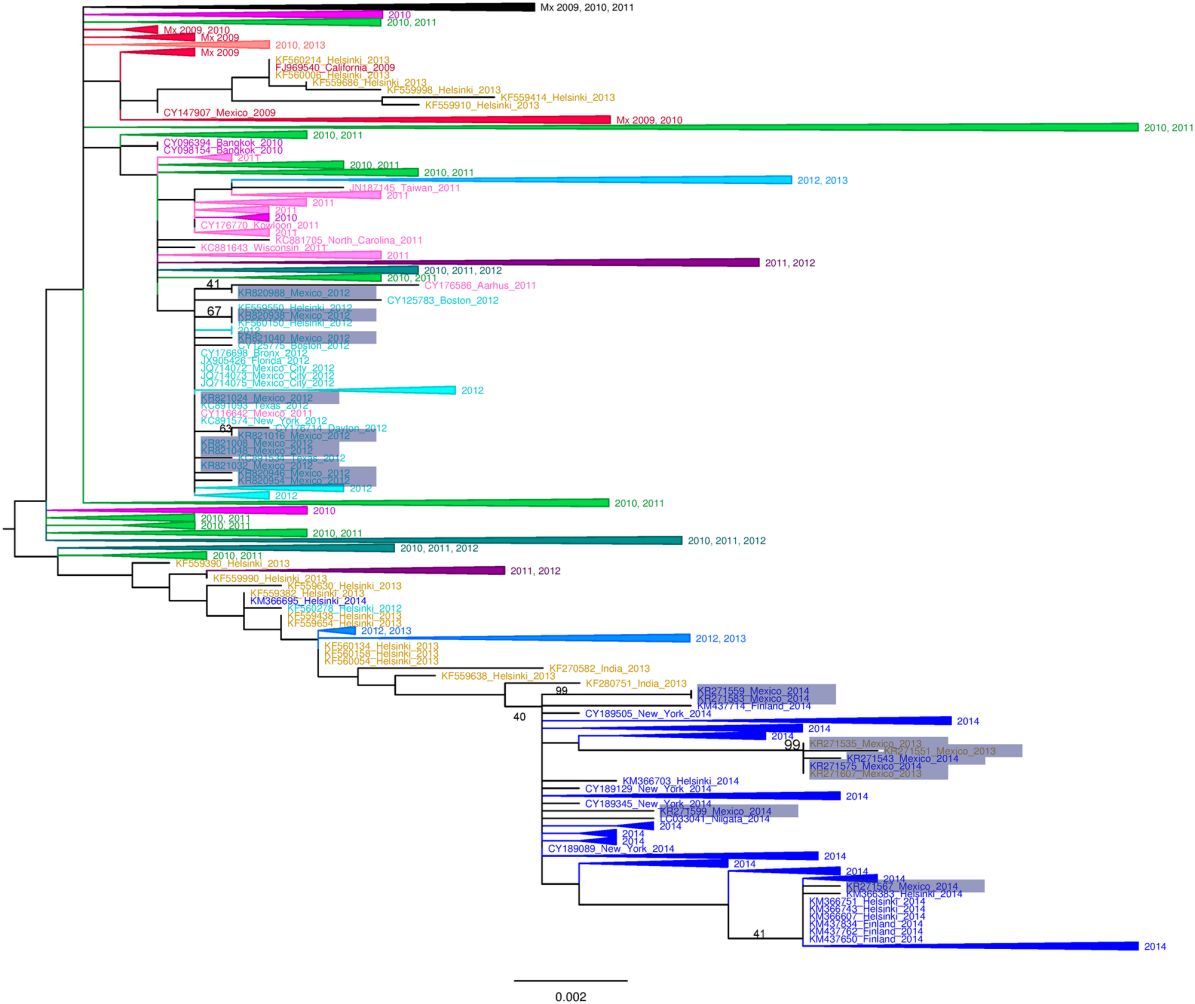
Maximum likelihood (ML) phylogenetic tree for the HA segment. ML trees from 1200–1300 A(H1N1)pdm09 viruses deposited in GenBank from 2009 to 2014 were produced with 1,000 bootstrap replicates, for the indicated genetic segments as explained in the Methods section. Phylogenetic tree included 7–17 sequences from 2012 (PB2, 10; PB1, 8; PA, 7; HA, 10; NP, 11; NA, 8; M, 17 and NS, 17), 3 sequences from 2013 and 7 sequences from 2014; obtained for this study. Red dots at nodes show branches with 50% bootstrap support leading to the 2014 sequences described in this work. Trees for the rest of the viral genome segments can be found in S1–S7 Figs (S1 Fig **NA**, S2 Fig **PB2**, S3 Fig **PB2**, S4 Fig **PA**, S5 Fig **M**, S6 Fig **NP**, S7 Fig **NS**). Colours for seasons: RED, 2009–2010; BRIGHT GREEN, 2010–2011; PURPLE, 2011–2012; BLUE SKY, 2012–2013; VIOLET, 2013–2014.

Sequences of segments HA, NP, NA, M and NS of the pandemic virus collected in the 2013–2014 influenza season clustered with the 2013–2014 sequences from New York, Helsinki, and Washington. However, polymerase complex sequences clustered with samples from 2010 and 2011. Analysis based on the HA gene showed that Mexican sequences could be divided into 3 groups: Group 1 (KR271535, KR271543, KR271551, KR271575, and KR271607) has 5 sequences forming a monophyletic group (99 bootstrap, Fig 1). These Mexican sequences clustered next to HA sequences of strains from New York (CY189313, CY189361, bootstrap value 66). These sequences share two substitutions in Signal Peptide of the immature HA at amino acid 7 and 15 (-11 V/I and -3 A/T). Group 2 (KR271559 and KR1571583) sequences clustered separately and shared a novel substitution in HA H138Q. Group 3 (KR271599 and KR271567) clustered with strains isolated in New York, Washington and Helsinki. One isolate (KR271567) shared mutation T474M with sequences from Helsinki and Finland principally. We mapped the amino acid substitutions occurring at the nodes of H1N1/2009 HA phylogeny, revealing amino acid changes at K163Q and A256T for 2013–2014 sequences as reported previously. Additionally, a cluster of 2 Mexican sequences was marked by substitution H138Q. Regarding NA, we observed 3 substitutions that marked sequences from 2013–2014; I34V, I321V and K432E. To determine whether individual sites were under positive selection, we used the mixed effects model of evolution (MEME) method. Only I34V was statistically significant (P = 0.05). Additionally we found substitutions that distinguished the three groups mentioned above with HA analyses. We observed substitution Q308L in group 1, while T452I was found in group 2 and the substitutions N449K, N386K and S82P were present on sequence KR271569 in group 3. For NS1 we detected the substitution L36I in group 1, the N127S in group 2 and finally the substitution K131E in group 3. Regarding PA we found the substitution I13V as a signature of the group 1 (statistically significant with P = 0.05), while in group 3 we detected two substitutions F35L and I459T. In PB1 we associated the substitution A374T with group 2 and M646I with group 3. In PB2 T676I was found in group 2[17]. Other substitutions were found in M1, M2, NS2 and NP but were similar to those reported in other countries (Table 2).

### Hemaglutinin (HA) molecular characterization

**Fig 2:**
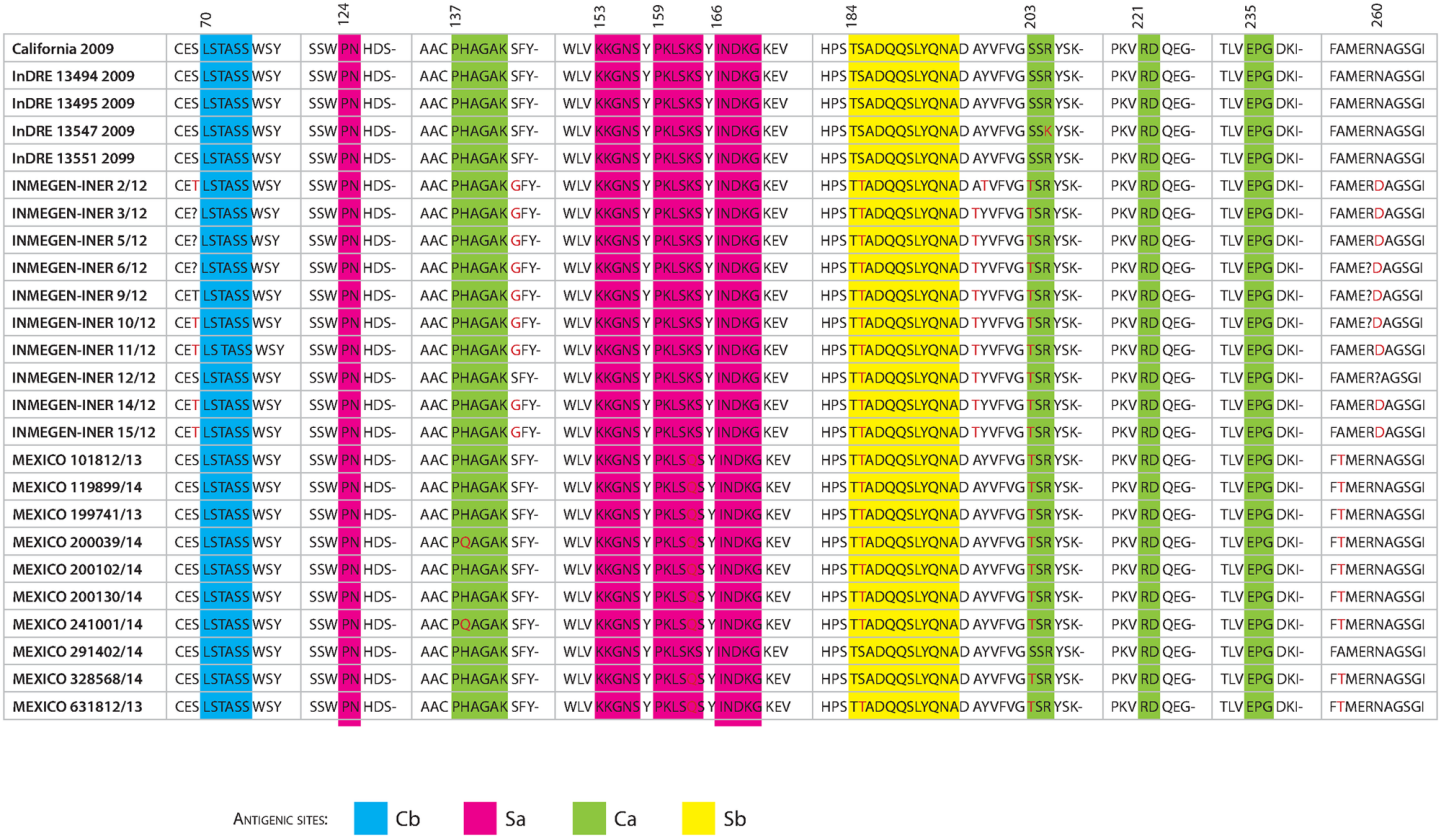
Analysis of detected substitutions at or beside antigenic sites of HA, of Mexican isolates from 2011–12 and 2013–14. The analysis of substitutions at HA was made using sequence California/07/2009 as reference to establish the changes at the antigenic sites and in their neighboring positions. The antigenic sites are shaded and identified by colours. Blue is for Cb site, pink is for Sa site, Green is for Ca site and yellow is for Sb site. The amino acids in red represent changes detected in sequences of isolates from 2011–12 and white represent the changes detected in isolates from 2013–14.

Mapping the substitutions to H1 antigenic sites showed different patterns of change through 2009–2014 winter seasons. HA substitutions S69T, S143G, A197T, N260D and V520A were the dominant mutations during 2011–2012 but not in the influenza season 2013–2014. Other mutations became dominant since 2011–2012, (S185T, E374K, S451N and E499K) and remain until 2013–2014, in addition to these substitutions, K163Q, A256T and K283E were dominant in Influenza virus from Mexico during 2013–2014. Importantly we detected the H138Q substitution, that is located on the Ca2 antigenic region of HA, a relevant immunogenic site (Fig 2). Six putative glycosylation sites on HA (Asn-Xaa-Ser/Thr) at positions 28, 40, 104, 304, 498 and 557 were predicted using NetNGlyc 1.0 server. This indicates that Mexican isolates from 2013–2014 maintain a glycosylation pattern similar to California 2009.

## Discussion

Influenza A viruses cause acute respiratory tract infections and represent a significant public health threat [18]. The outbreak strain of swine-origin influenza A/H1N1 virus infection in 2009 is still circulating during the winter season in many countries, and may cause severe pulmonary illness in susceptible individuals [4, 19]. Therefore continuous analysis of the entire genome of these viruses will provide comprehensive information of its molecular evolution in order to maintain effective prevention measures for public health. It has been extensively demonstrated that pandemic A/H1N1 influenza virus contains a mixture of segments, including HA, NP, PB1, PB2, PA and NS from a triple reassortant virus isolated in north America and segments NA and M from the Eurasian swine influenza virus [20, 21].

Due to the differential mutation rates of each viral segment, molecular epidemiology surveillance is important to detect antigenic variations among circulating influenza strains, which can modify the pathogenicity and antiviral resistance patterns of these viral strains.

In this study we sequenced the entire genome of pandemic A/H1N1 strains isolated from patients in a reference Hospital in Mexico City (INER) in different years and we compared these sequences with consensus sequences in order to detect mutations that might be associated with viral evolution or might influence the antigenicity of the virus. It is important to mention that the sequences were obtained directly from clinical samples, in order to avoid in vitro artificial selection.

We found a clear variation of the virus in Mexico from the 2011–2014 season due to different markers and in accordance with previous reports [22, 23]. Mutations V344M and I354L of PB2 and N321K of PA, I397M, I435T of PB1, S498N of NP, N44S, V241L, N369K of NA V80I of M1, L90I of NS1 and S185T, S203T, E374K and S451N of HA appeared together during the evolution of influenza virus in Mexico. However we found other unique substitutions in PB2 G644R and T676I, PB1 A374T, HA H138Q, NA Q308L, and L36I in NS1 of some Mexican strains that could indicate geographical divergence. These findings should be confirmed with more sequences obtained in other regions of the country. Recently a very extensive analysis of sequences of influenza virus in a post-pandemic era showed that different geographical regions generated local epidemic peaks and might act as a potential seed of local virus. Further studies would be required in order to determine which of them are fixed in the next generation of influenza virus in Mexico. It is known that influenza viruses undergo positive selection during the process of cross-species transmission and during the initial stages of a human outbreak, followed by purifying (negative) selection, when viruses adapted to the new human host in late epidemic [24–26]. In this context we still observed positive selection of substitutions of NA I34V and PA I13V in winter season 2013–2014.

Our data indicate that the polymerase complex appears to have different evolutionary dynamics; probably due to differences in the evolutionary rate among the viral segments. This might also be a reflection of differences in selective pressure once the virus is in the infected host; regarding the rest of the viral genome segments, all the Mexican isolates from season 2013–2014 clustered with sequences from New York and Helsinki.

Mexican sequences from 2013–2014 belonged to clade 6B characterized by D97N, K163Q, S185T, K283E and A256T substitutions (Fig 1). However a different branch was observed with 2 sequences presenting specific substitutions in all segments, including the substitution H138Q which lied in antigenic site in HA, These results suggest that strains could appear temporarily, with different molecular characteristics and potentially with antigenic or pathogenic implications.

A recently significant change occurred in virus from 2013 to 2014, the substitution K163Q were predominant from these years until now and has been associated with binding prevention of antibodies (Abs) elicited in a larger number of middle-aged humans all over the world [27].

In this study we identified 13 novel substitutions with important biological effects, including virulence, T cell epitope presented by MHC and potential roles in host specificity shifts and it should be noted that although these mutations have not been particularly characterized, they are found to be close to or belong to regions of biological importance previously (Table 2).

Substitution PB2-R591P is on a position where R has been implicated in a more efficient replication of pandemic H1N1 viruses in mammals [28] while the effect of proline is not known. PB1-S704T is part of the H-2K^b^ epitope and might affect the efficiency of antigen processing during cytotoxic T lymphocyte (CTL) response [29, 30]. E16A is in the N-terminal ectodomain of M2 at the outside of the viral membrane near to the disulfide bridges connecting the M2 tetramer 18235503] but its specific effect has not been particularly characterized yet [31].

Other novel substitutions in this report are involved in **VOI**, such as PA at position T162P[32, 33]; **BSL** in PB2 at V640M [34] and in PA at L109F[32, 33]; **VOI**/**BNA** in NP at G356R [35] and **BSL/VOI** in NS1 at Q63K [36].

Regarding previously observed substitutions, some have been computationally predicted to be linked to host specificity, such as PB2 V613A [37]. Other roles of residues include **VOI**, I13V in PA [38], S482N in NP[35]; G18S and A33T in M1; A122T in NS1 [39] [40]. **BSL**, F35L in PA [41].

It was observed before that some substitutions might have more than one biological function, such as HA H25R and E373G, which are implicated in Ab’s recognition, **BSL**, **VOI**, and **BHP** [42, 43]. NA D199N is also associated with antigenic drift [44, 45]; **BSL/VOI** in PB2 G644R [46]; PA T151N and E623D [32, 33, 47]; NA, V177I [45]; NS1, L36I, Q63K, N127S [40, 48]. **BHP** in NA T452I involved in **BSL** [49] and, in NS1 S74T [40, 48], related to **BSL** and **VOI**. **BNA** in NP G356R [35] and NS1, G45 related with **VOI** [50]. All of these sites have been described by crystalographic or computational analysis (Table 2).

In HA we observe changes that could affect immunogenicity of the influenza virus; sequences from 2015 to 2016 had additional mutations (S162N) in antigenic site (Sa) and together with the substitution I276T are defining a new clade 6B.1 [21, 22]. Importantly S162N may confer an additional glycosylation site in HA that could affect even more the immunogenicity of influenza virus in the future.

Our results indicate that the pandemic influenza virus changes through the seasons and show how the use of whole genome sequences are able to provide a deeper understanding of virus evolution through time.

## Supporting information

S1 Table Accession numbers of sequence used as reference and accession numbers of sequences from this study. (DOCX)

S1 Fig Phylogenetic tree for NA segment. (TIF)

S2 Fig Phylogenetic tree of PB1 segment. (TIF)

S3 Fig Phylogenetic tree of PB2 segment. (TIF)

S4 Fig Phylogenetic tree of PA segment. (TIF)

S5 Fig Phylogenetic tree of M segment. (TIF)

S6 Fig Phylogenetic tree of NP segment. (TIF)

S7 Fig Phylogenetic tree of NS segment. (TIF)
