# Supplementary material for: Molecular features of influenza A (H1N1)pdm09 prevalent in Mexico during winter seasons 2012-2014
Source: PLoS One. 2017 Jul 10;12(7):e0180419. doi: 10.1371/journal.pone.0180419 (PMC5503254; doi:10.1371/journal.pone.0180419)
Supplement: S1 Table — (DOCX) [file pone.0180419.s001.docx]

**Supplementary material**

**Table S1. Accession numbers of sequence used as reference and accession numbers of sequences from this study**

| Gene | Accession numbers A/California/07/2009(H1N1)/2009 | Accession number 2012 sequences | Accession number 2013-2014 sequences |
| --- | --- | --- | --- |
| PB2 | KC781783 | KR820943  KR821005  KR820959  KR821037  KR821013  KR821001  KR820977  KR821021  KR821045  KR820985  KR820935  KR820993  KR820951  KR821029  KR820928  KR820967 | KR271532  KR271548  KR271604  KR271540  KR271556  KR271564  KR271572  KR271580  KR271588  KR271596 |
| PB1 | KC781787  FJ969531 | KR820944  KR821006  KR821014  KR820978  KR821022  KR821046  KR820986  KR820936  KR820952  KR821030  KR820929  KR820968 | KR271533  KR271549  KR271605  KR271541  KR271557  KR271565  KR271573  KR271581  KR271589  KR271597 |
| PA | KC781786 | KR820945  KR821007  KR821039  KR820979  KR821023  KR821047  KR820987  KR820937  KR820953  KR821031  KR820969 | KR271534  KR271550  KR271606  KR271542  KR271558  KR271566  KR271574  KR271582  KR271590  KR271598 |
| HA | KF009554  FJ981613 | KR820946  KR821008  KR820962  KR821040  KR821016  KR820980  KR821024  KR821048  KR820988  KR820938  KR820996  KR820954  KR821032 | KR271535  KR271551  KR271607  KR271543  KR271559  KR271567  KR271575  KR271583  KR271591  KR271599 |
| NP | FJ969536  GQ338390 | KR820974  KR820947  KR821009  KR820963  KR821041  KR821002  KR820981  KR821025  KR821049  KR820989  KR820939  KR821033  KR820931  KR820970 | KR271536  KR271552  KR271608  KR271544  KR271560  KR271568  KR271576  KR271584  KR271592  KR271600 |
| NA | KF009555  GQ377078 | KR820948  KR821010  KR821042  KR820982  KR821026  KR821050  KR820990  KR820940  KR820956  KR821034  KR820932  KR820971 | KR271537  KR271553  KR271609  KR271545  KR271561  KR271569  KR271577  KR271585  KR271593  KR271601 |
| M | FJ966975  FJ969527 | KR820975  KR820949  KR821011  KR820965  KR821043  KR821019  KR821003  KR820983  KR821027  KR821051  KR820991  KR820941  KR820999  KR820957  KR821035  KR820933  KR820972 | KR271538  KR271554  KR271610  KR271546  KR271562  KR271570  KR271578  KR271586  KR271594  KR271602 |
| NS | KC781782 | KR820976  KR820950  KR821012  KR820966  KR821044  KR821020  KR821004  KR820984  KR821028  KR821052  KR820992  KR820942  KR821000  KR820958  KR821036  KR820934  KR820973 | KR271539  KR271555  KR271611  KR271547  KR271563  KR271571  KR271579  KR271587  KR271595  KR271603 |
