# Supplementary figures and images for: Molecular features of influenza A (H1N1)pdm09 prevalent in Mexico during winter seasons 2012-2014
Source: PLoS One. 2017 Jul 10;12(7):e0180419. doi: 10.1371/journal.pone.0180419 (PMC5503254; doi:10.1371/journal.pone.0180419)

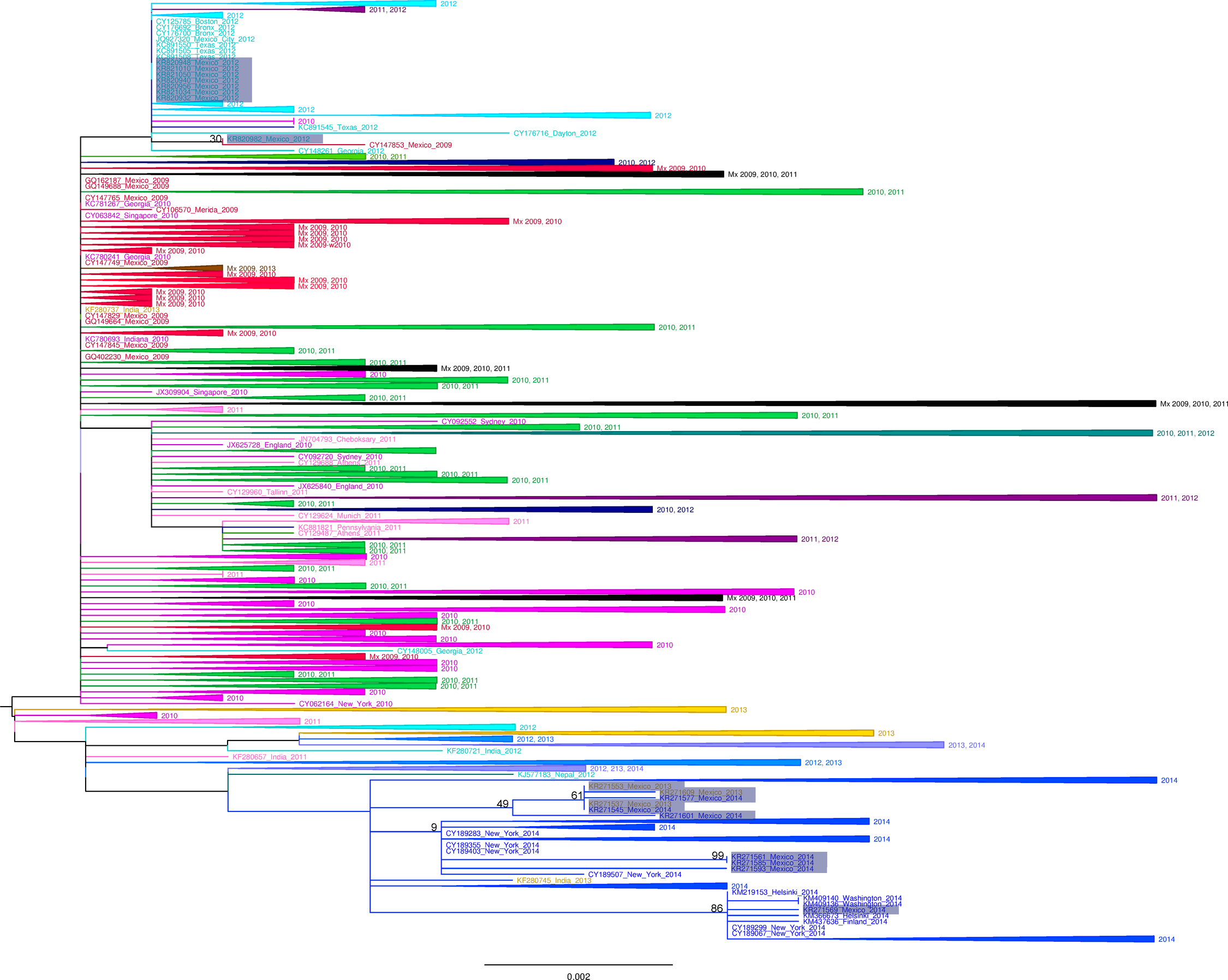

Supplement: S1 Fig — (TIF) [file pone.0180419.s002.tif]

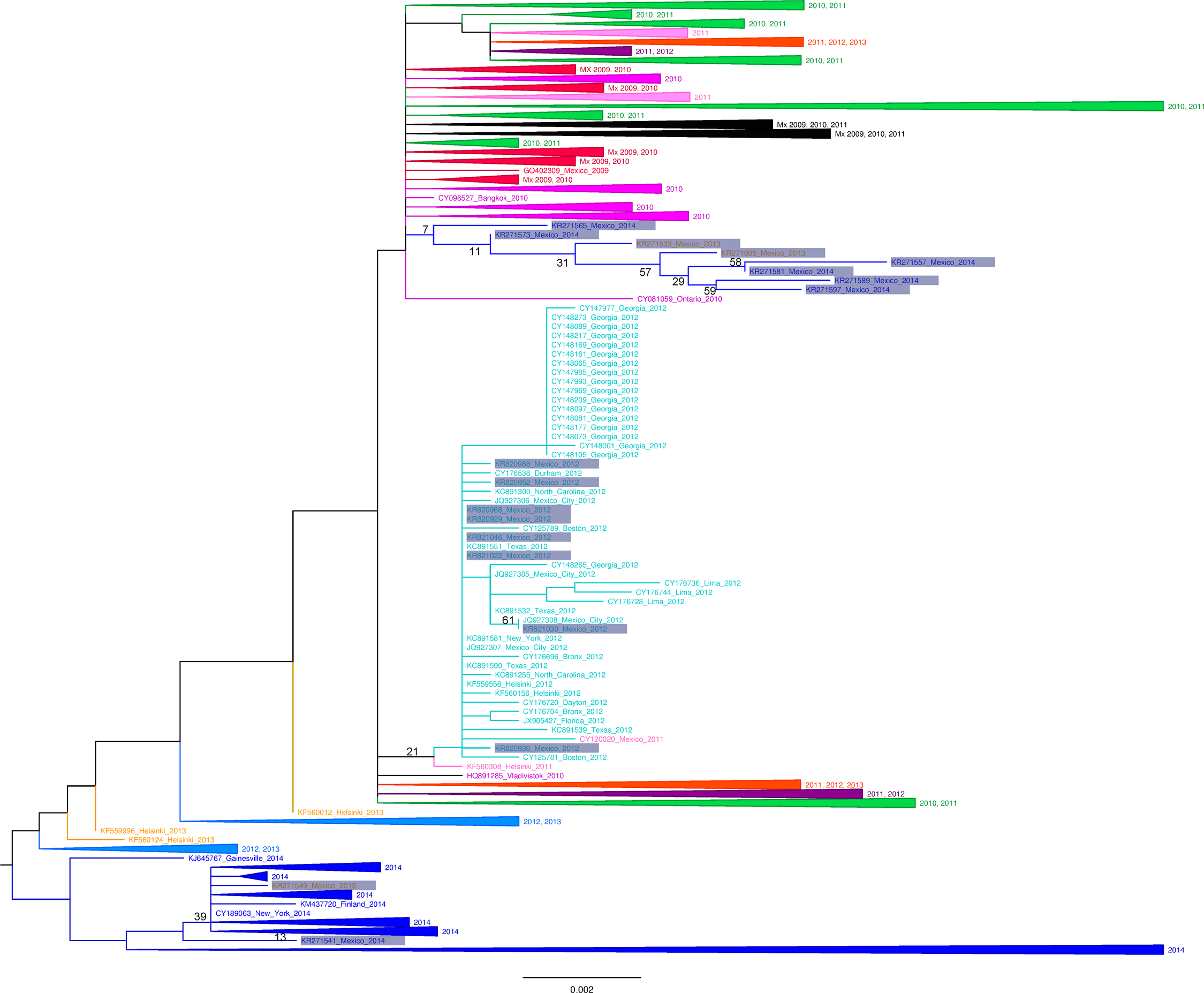

Supplement: S2 Fig — (TIF) [file pone.0180419.s003.tif]

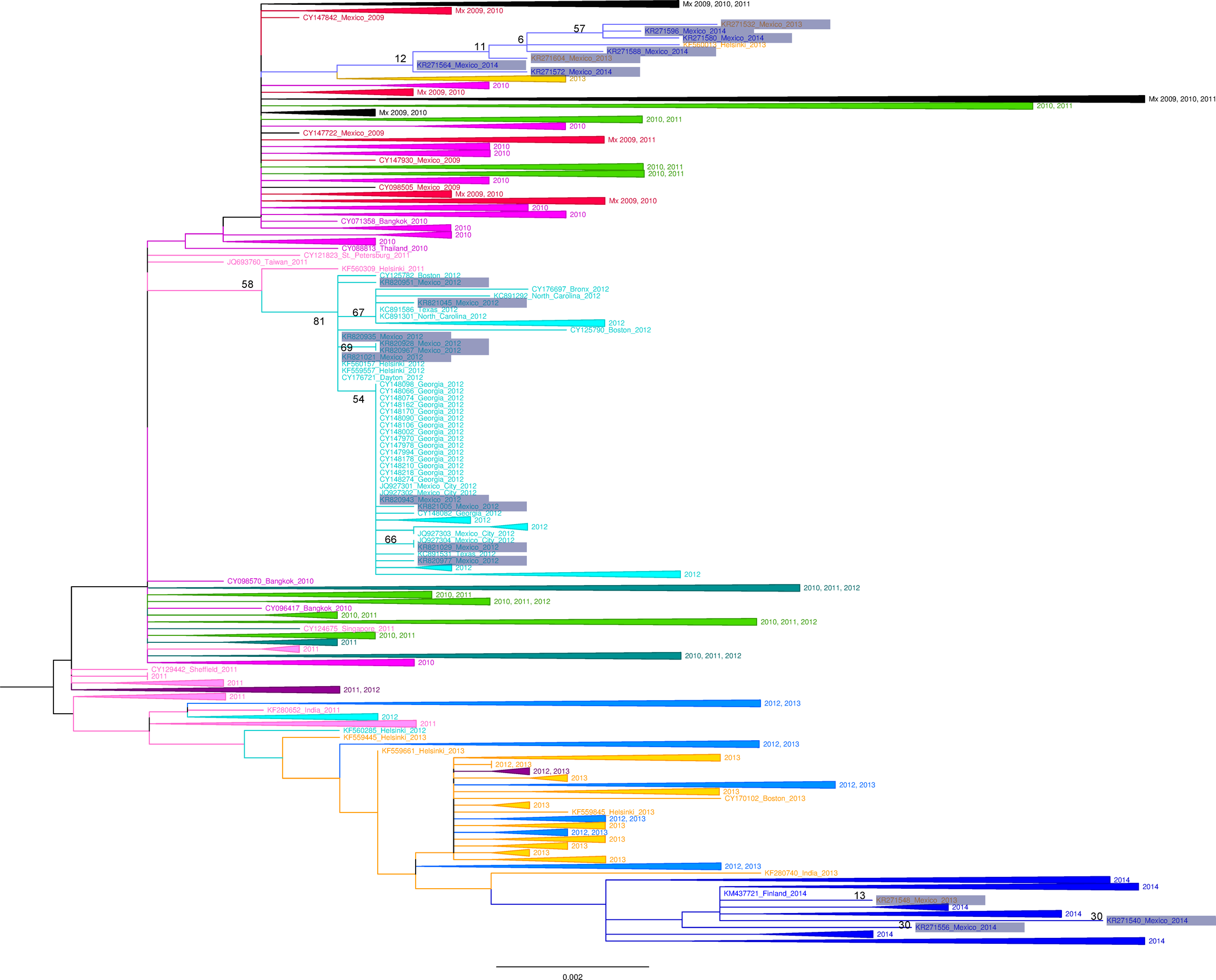

Supplement: S3 Fig — (TIF) [file pone.0180419.s004.tif]

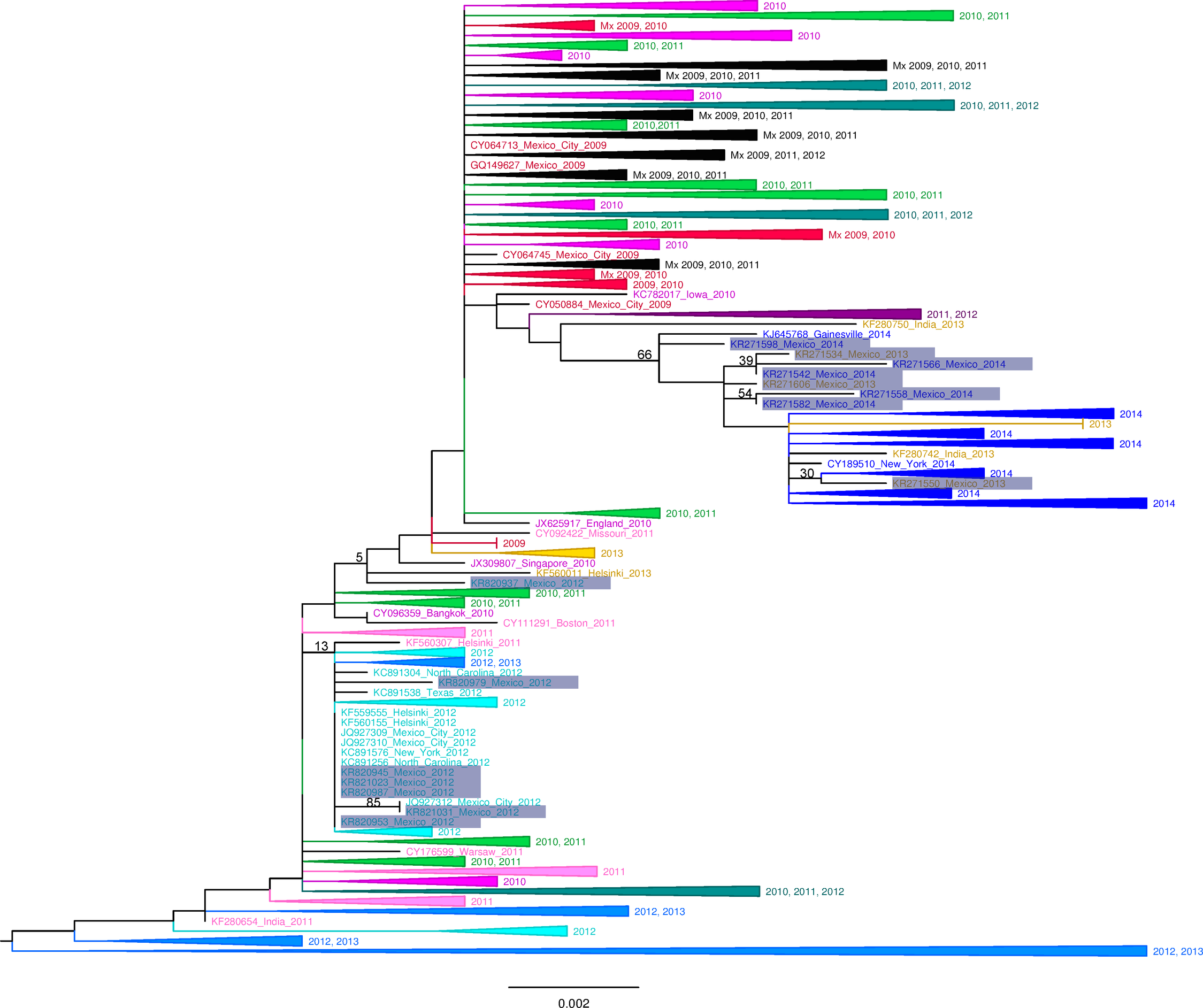

Supplement: S4 Fig — (TIF) [file pone.0180419.s005.tif]

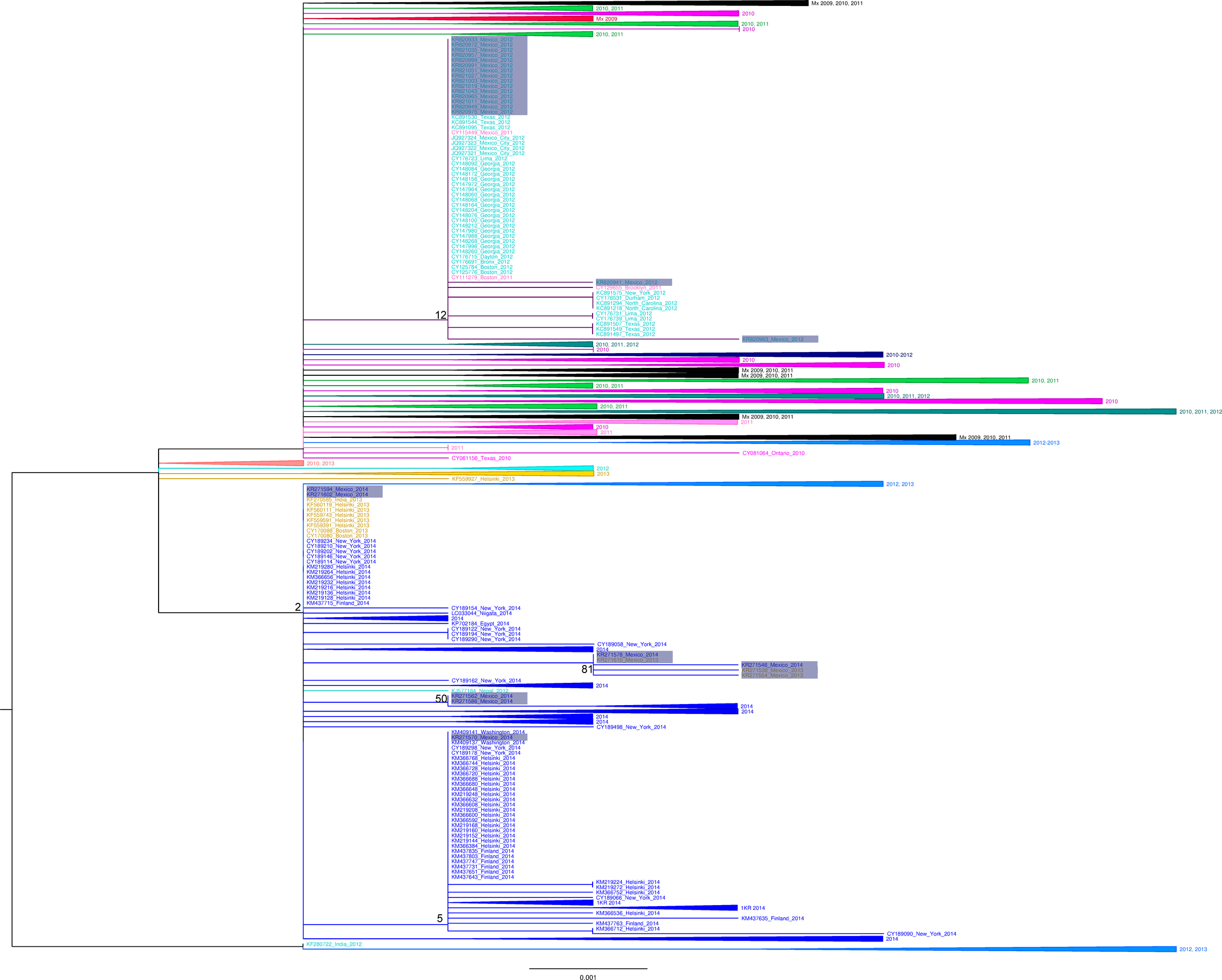

Supplement: S5 Fig — (TIF) [file pone.0180419.s006.tif]

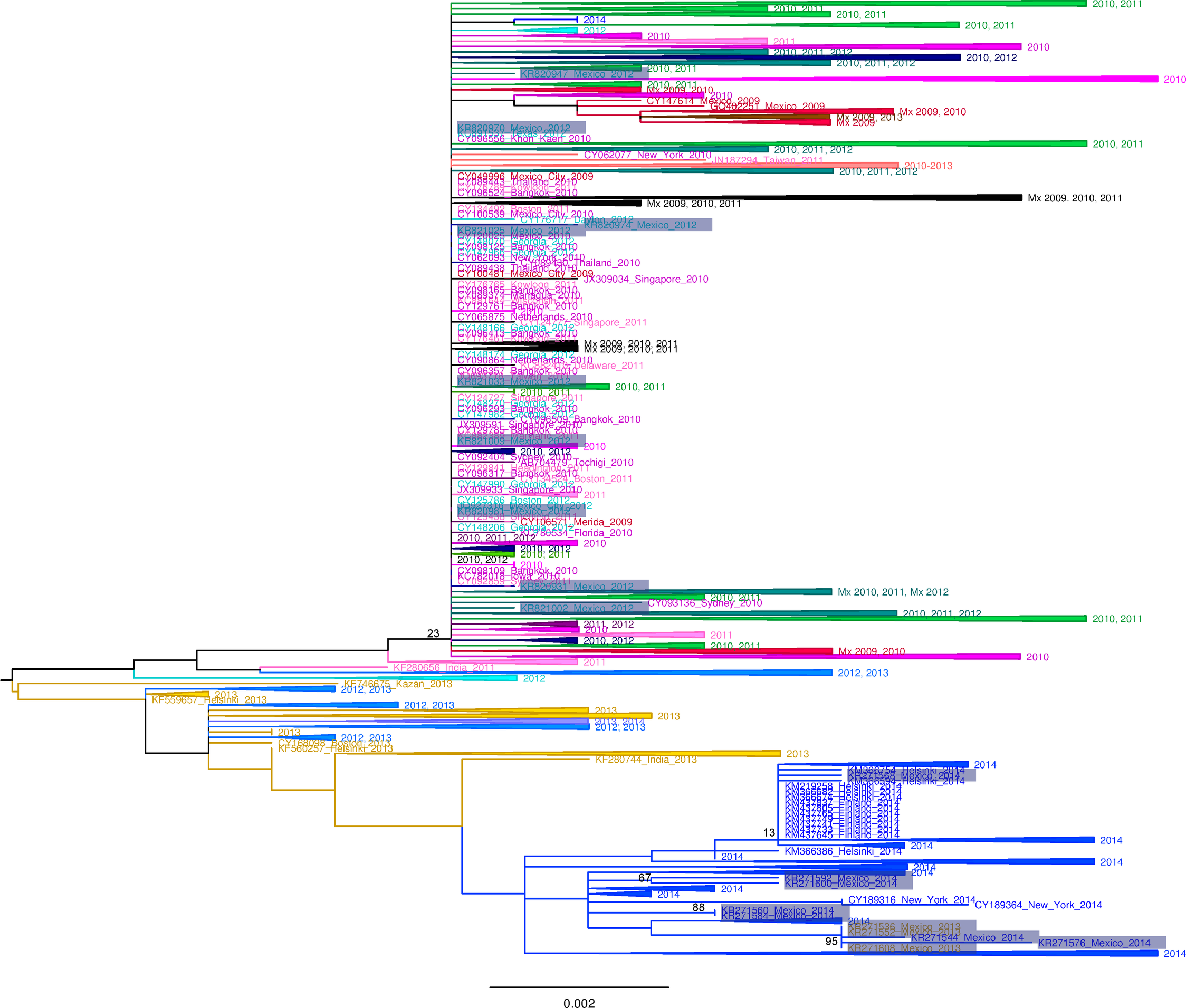

Supplement: S6 Fig — (TIF) [file pone.0180419.s007.tif]

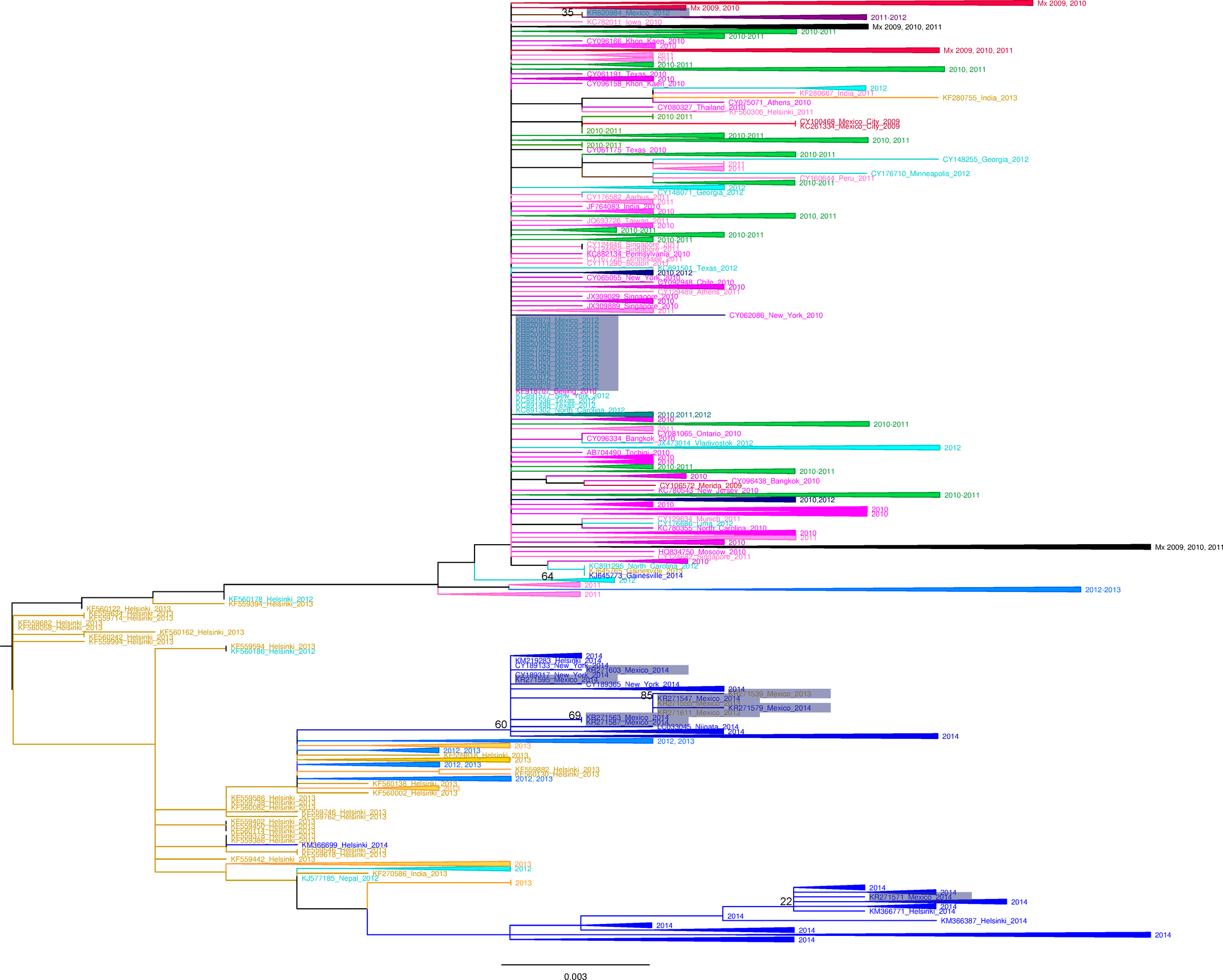

Supplement: S7 Fig — (TIF) [file pone.0180419.s008.tif]
